# Comparative genomic analysis of evolutionarily conserved but functionally uncharacterized membrane proteins in archaea: Prediction of novel components of secretion, membrane remodeling and glycosylation systems

**DOI:** 10.1016/j.biochi.2015.01.004

**Published:** 2015-01-09

**Authors:** Kira S. Makarova, Michael Y. Galperin, Eugene V. Koonin

**Affiliations:** National Center for Biotechnology Information, National Library of Medicine, National Institutes of Health, Bethesda, MD 20894, USA

**Keywords:** Archaeal genomes, arCOGs, Membrane proteins, Gene neighborhoods

## Abstract

A systematic comparative genomic analysis of all archaeal membrane proteins that have been projected to the last archaeal common ancestor gene set led to the identification of several novel components of predicted secretion, membrane remodeling, and protein glycosylation systems. Among other findings, most crenarchaea have been shown to encode highly diverged orthologs of the membrane insertase YidC, which is nearly universal in bacteria, eukaryotes, and euryarchaea. We also identified a vast family of archaeal proteins, including the C-terminal domain of N-glycosylation protein AglD, as membrane flippases homologous to the flippase domain of bacterial multipeptide resistance factor MprF, a bifunctional lysylphosphatidylglycerol synthase and flippase. Additionally, several proteins were predicted to function as membrane transporters. The results of this work, combined with our previous analyses, reveal an unexpected diversity of putative archaeal membrane-associated functional systems that remain to be functionally characterized. A more general conclusion from this work is that the currently available collection of archaeal (and bacterial) genomes could be sufficient to identify (almost) all widespread functional modules and develop experimentally testable predictions of their functions.

## 1. Introduction

Semipermeable lipid bilayer membranes are a major hallmark of all living cells. Despite striking differences in membrane lipid structure between archaea and bacteria [1–3], these two domains of cellular life share many membrane proteins, including several components of ATP synthase, NADH dehydrogenase, Sec and Tat protein translocase complexes and numerous transporters [4–6]. The total shares of (predicted) membrane proteins encoded in the genomes of bacteria and archaea are also similar, ranging from 20 to 30% [7–9]. Despite the substantial progress in understanding the organization of biological membranes and membrane proteins over the past several decades, membrane proteins remain notoriously difficult to study both experimentally and computationally. These difficulties stem primarily from the fact that most of the membrane proteins are highly hydrophobic and therefore insoluble in aqueous media. This property is also reflected in the sequences of membrane proteins that are characterized by high (often up to 90%) content of hydrophobic amino acid residues which typically form clusters corresponding to transmembrane helices (TMs) [10,11]. This feature is successfully exploited for membrane protein prediction [9,12–14] but makes it difficult to study such proteins using standard methods of sequence comparison. Another obstacle is that due to the weak constraints on the amino sequences, beyond the required hydrophobicity, membrane proteins often evolve faster than soluble globular proteins [15,16]. However, despite this relatively fast evolution, in many cases, some sequence similarity is observed between non-homologous membrane proteins, purely because of the presence of compositionally similar TMs. This “promiscuity” complicates the classification and functional prediction for membrane proteins. As a consequence of the structural features of membrane proteins reflected in their mode of evolution, the annotation of the membrane proteins in sequenced genomes is often poor, even for some components of evolutionarily conserved membrane complexes. In many cases, functional annotation of membrane proteins could benefit from taking into account the genomic context of the respective genes, under the “guilt by association” approach that is well known and highly productive in microbial genomics [17–19]. The success of this approach critically depends on precise identifications of orthologous genes in all analyzed genomes. Over the last several years, our group has been maintaining the arCOG database in which the clusters of orthologous genes from archaea are manually curated with respect to both membership and predicted function [5,20]. Here, using sensitive sequence comparison methods and gene context analysis, we investigate the arCOGs that include functionally uncharacterized proteins that are projected to the Last Archaeal Common Ancestor (LACA) and for which membrane localization is confidently predicted. We predict either general function or a specific cellular process for the majority of these proteins and identify several putative novel archaeal secretion or membrane remodeling systems. These predictions are intended to guide further experiments with these important archaeal proteins.

## 2. Materials and methods

### 2.1. Sequence data

A 2014 update of the archaeal clusters of orthologous genes (arCOGs) was used to annotate protein-coding genes in 168 archaeal genomes [5], available at the NCBI FTP site (ftp://ftp.ncbi.nih.gov/pub/wolf/COGs/arCOG/). Genome sequences were downloaded from the NCBI FTP site (ftp://ftp.ncbi.nlm.nih.gov/genomes/Bacteria/). Phyletic patterns (patterns of presence or absence of given proteins families in the analyzed genomes) were derived from the respective arCOGs assignments.

### 2.2. Sequence analysis

Iterative profile searches with the PSI-BLAST [21], with a cut-off e-value of 0.01, and composition based-statistics and low complexity filtering turned off, were used to search for distantly similar sequences in NCBI's non-redundant (NR) database. Additionally, another sensitive method for remote sequence similarity detection, HHpred, was used with default parameters [22]. Multiple sequence alignments were constructed using MUSCLE [23]. Protein secondary structure was predicted using Jpred [24].

For each analyzed gene, the Pfam [25] identifiers and COG [26] numbers as well as the corresponding annotations were assigned using the RPS-BLAST program and the CDD database of profiles [27].

Transmembrane segments were predicted using the TMHMM v. 2.0c program with default parameters [9]. Signal peptides were predicted using the SignalP v. 4.1c program; the union of the three predictions (gram-negative, gram-positive and eukaryotic models) was used [28].

## 3. Results

We used the results of the reconstruction of the gene complement of LACA [5] to select 978 arCOGs that project to LACA with the probability of 90% or greater. In 105 of these arCOGs (~11%), two or more transmembrane (TM) helices were predicted (Supplementary Table 1). Proteins with a single predicted TM were not considered in order to exclude false positives as well as secreted proteins in which the signal peptide is often predicted as a TM. For each of these 105 arCOGs, we checked annotations of the respective proteins in the RefSeq database [29] and selected 15 families in which the majority of proteins were annotated as “hypothetical” (Table 1). These arCOGs were then analyzed on a case-by-case basis in order to characterize each family in detail and attempt to predict the function of these conserved but uncharacterized proteins. It should be noted that the set of 15 arCOGs is the low bound of the uncharacterized conserved membrane proteins because, although many other proteins in the 105 arCOGs set have some general function annotation, they have never been studied experimentally and their actual function or specificity might be different from the current assignment.

### 3.1. arCOG02673 and other homologs of OxaA/SpoJ/YidC insertase

**Fig. 1:**
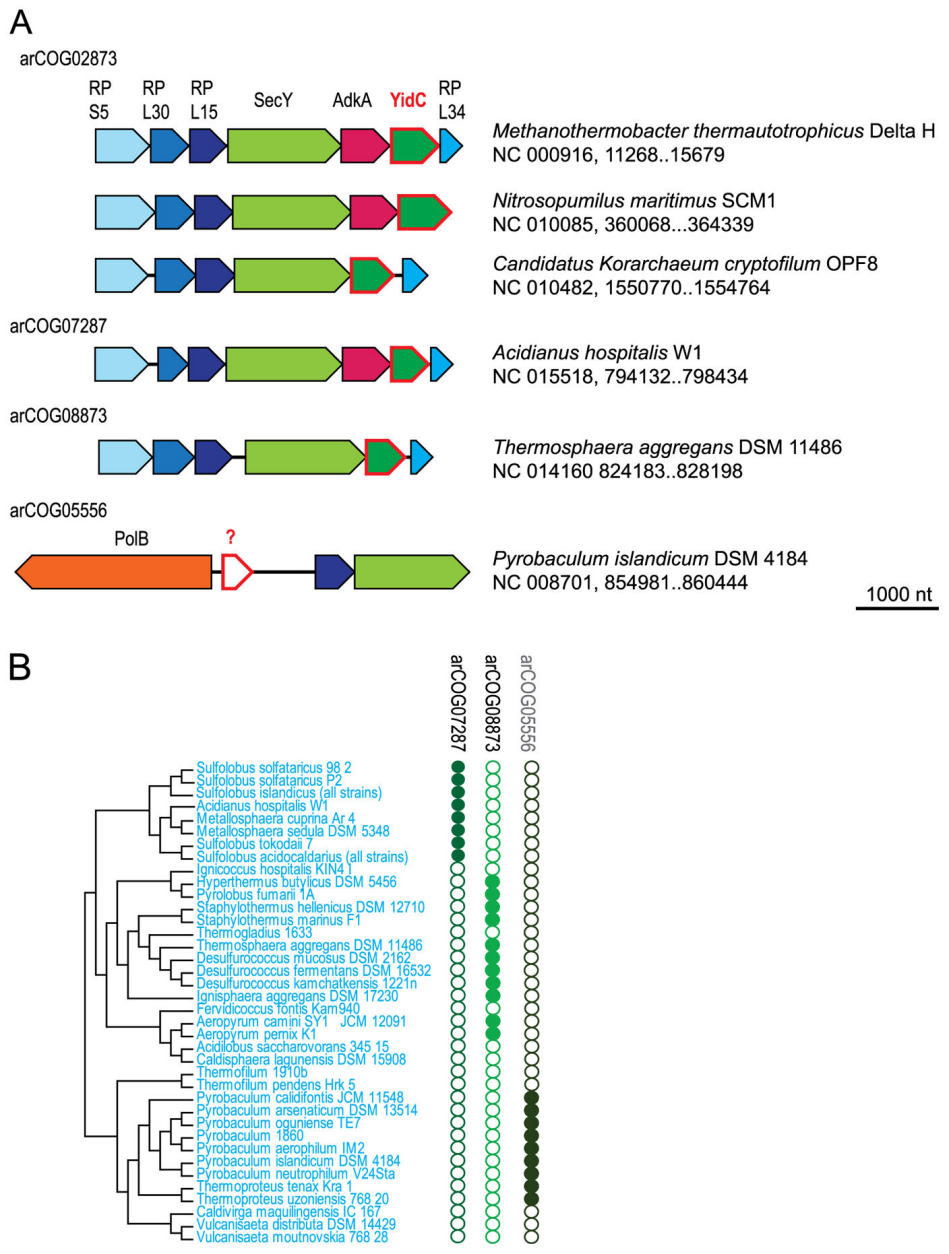
Comparative genomic analysis of the YidC family in crenarchaea. A. Gene neighborhoods of predicted *yidC* genes in archaea. For each arCOG gene neighborhoods for representative organisms are shown. Genes are shown by block arrows with the length roughly proportional to the size of the corresponding gene. Homologous genes are indicated by the same color. The annotated arCOGs are indicated above the respective arrows. The arCOG05556 gene is represented by a white arrow to show that the evidence for it being the YidC subunit is weak. Abbreviations: RP S5 – ribosomal protein S5, RP L30 – ribosomal protein L30, RP L15 – ribosomal protein L15, SecY – preprotein translocase subunit SecY, RP L34 – ribosomal protein L34E, AdkA – archaeal adenylate kinase, PolB – DNA polymerase elongation subunit (family B). B. Phyletic pattern of predicted YidC subfamilies in different archaeal lineages. Phyletic patterns for the indicated arCOG families (filled circles show presence and empty circles show absence of the respective COG members) are superimposed over the phylogenetic tree of crenarchaea. The tree topology is based on the phylogeny of concatenated ribosomal proteins [81].

YidC is an essential protein present in almost all bacteria and eukaryotes, where it functions in mitochondria and chloroplasts [30]. YidC is a membrane protein containing 5 or 6 TMs that catalyzes cotranslational membrane insertion of proteins in cooperation with the Sec translocase. Homologs of YidC in euryarchaea have been identified over a decade ago [31]. Nevertheless, these proteins are still annotated as “hypothetical” in most databases and a vast majority of complete genomes. In the arCOG database, arCOG02673 (COG1422, pfam01956, a typical representative is TK1516 from *Thermococcus kodakarensis*) corresponds to YidC and includes eury-, thaum- and korarachaeal sequences (Supplementary Table 2). This assignment that was initially based on sequence conservation is supported by genomic neighborhood analysis. The archaeal YidC homolog is encoded in a conserved gene neighborhood that is represented in most euryarchaea, thaumarchaea, korarchaea and nanoarchaea and is located in the vicinity of the *secY* gene which encodes the universally conserved membrane subunit of the Sec translocase complex (Fig. 1A). YidC has been shown to interact with SecY during cotranslational insertion of proteins into membranes, including such essential membrane proteins as *a* and *b* subunits of the ATP synthase and the twin arginine translocase TatC ([30] and references therein). In addition, YidC interacts with the ribosome and plays an important role in stress response [30], which is compatible with the colocalization of the *yidC* gene with ribosomal protein genes in most archaeal genomes (Fig. 1A).

Considering the presence of SecY and other Sec translocase subunits in all archaea, it seemed surprising that YidC had not been identified in crenarchaea [32]. In an attempt to identify YidC orthologs in these genomes, we analyzed the *secY* gene neighborhoods and detected two uncharacterized arCOGs, namely arCOG07287 (a typical representative is SacN8_02775 from *Sulfolobus acidocaldarius*) and arCOG08873 (a typical representative is DKAM_1151 from *Desulfurococcus mucosus*) that are located in a similar gene context in many Sulfolobales and Desulfurococcales. Using the sequences of these proteins as queries for PSI-BLAST search, we identified several additional homologous proteins that had not been assigned to the respective arCOGs by the automatic clustering procedure (Fig. 1B and Supplementary Tables 2 and 3). For the arCOG07287, HHpred search initiated with Ahos_0869 protein from *Acidianus hospitali* identified weak sequence similarity to YidC (with 82.5% probability for COG0706, bacterial YidC profile). The arCOG08873 proteins are more diverged and HHpred failed to detect similarity with COG0706. Nevertheless, the best hit for this family (e.g., for the query protein Shell_1420 from *Staphylothermus hellenicus*) is the profile of COG1422 (corresponding to arCOG02673) with the probability of 79.68%. These observations suggest that members of arCOG07287 and arCOG08873 are crenarchaeal orthologs of YidC. We could not confidently identify a YidC homolog in several members of Desulfurococcales and all Thermoproteales. In the latter case, the best candidate is arCOG05556 (a typical representative is PAE2104 from *Pyrobaculum aerophilum*) which includes proteins with 3 predicted TMs, is conserved in most of the Thermoproteales and is located in the vicinity of the *secY* gene in some of them (Fig. 1 and Supplementary Table 3). Thus, this analysis shows that a great majority if not all of the archaea encode orthologs of bacterial and eukaryotic YidC proteins, which can be confidently predicted to function as insertases similarly to the YidC function in bacteria and eukaryotic organelles.

### 3.2. arCOG01314 and arCOG02884 are components of a predicted secretion or membrane remodeling complex

arCOG01314 (N-terminal part of which is known as DUF4350, pfam14258; a typical representative is TON_1832 from *T. onnurineus*) is represented in a wide range of archaea (Table 1) and in several bacteria. The proteins of this family contain a predicted signal peptide and a single C-terminal TM, indicating that they are anchored in the membrane and possess a large extracellular N-terminal domain. An HHpred search for *Pyrococcus abyssi* protein PAB1295 revealed similarity with GldG, the substrate-binding subunit of a gliding motility-associated ABC transporter (profile TIGR03521, probability 99.24%) and additional homologs including pfam08532, β-galactosidase trimerisation domain (probability 97.06%), roughly corresponding to the middle domain of the β-galactosidase from *Thermus thermophilus*, which has a flavodoxin fold [33]. This observation implies that arCOG01314 proteins might form homomeric or heteromeric complexes. The gliding motility system is currently classified as a Type IX secretion system and has been implicated not only in gliding motility but also in secretion of large proteins such as cell-surface adhesins SprB, RemA and chitinase ChiA [34–36].

**Fig. 2:**
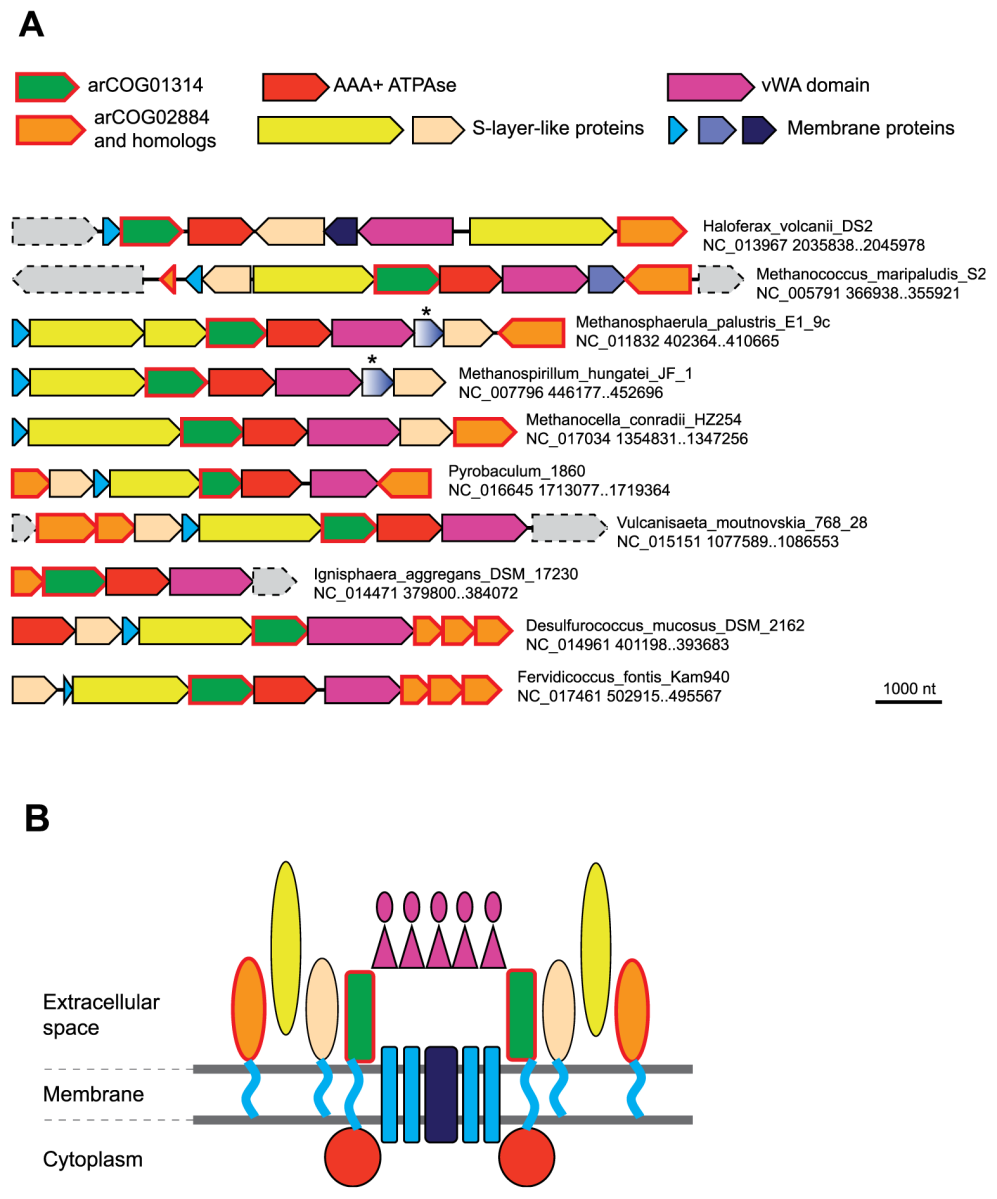
Neighborhood analysis and a model of putative novel secretion system associated with arCOG01314. A. Gene neighborhoods of arCOG01314 for several representative organisms. Designations are as in Fig. 1. The details for each family are described in Table 2. Specific arCOGs numbers present in each neighborhood are provided in the Supplementary Table 3. B. A hypothetical model of the putative novel secretion system. The cartoon is based on the features of each family described in Table 2. The green shape represents a membrane-anchored protein of arCOG01314. Oval yellow, orange and pale orange shapes represent three distinct families of proteins with Ig-like region. Purple shapes represent extracellular proteins containing vWa and Ig-like domains. Blue and sky blue shapes represent two distinct families of membrane proteins. The stoichiometry and positions of the subunits are not known and should be the subject of further experimental investigation.

The majority of the predicted operons containing arCOG01314 genes additionally encompass a gene encoding a secreted protein containing a von Willebrand factor type A (vWA) domain and a gene for an AAA +-type ATPase of COG0714 (Fig. 2A). The ATPase of this family and vWA domain-containing proteins also co-occur in other gene contexts in both bacteria and archaea, suggesting that this pair of proteins represents a distinct functional module (EVK, KSM unpublished). vWA domain-containing proteins have been extensively studied in eukaryotes and are known to interact with other domains to form multi-subunit complexes involved in a variety of cellular functions such as basal membrane formation, cell migration, cell differentiation, adhesion, signaling, and chromosomal stability [37,38]. The vWA-domains fused to AAA + ATPases function as Mg^2+^ and Co^2+^ chelatases that are involved in protoporphyrin IX biosynthesis; these chelatases are represented in bacteria, archaea and chloroplasts [39]. Additionally, vWA-domain proteins have been shown to associate with the FtsZl1 family and hence predicted to participate in membrane remodeling and vesicle formation [40,41]. In *S. acidocaldarius*, a distinct vWA-domain containing protein, Saci_1211 (ArnB; arCOG2900), is involved in repression of the archaellum operon [42].

Other components of the arCOG01314-encoding genome neighborhood include a number of membrane and secreted proteins some of which contain predicted extracellular immunoglobulin-like domains and predicted S-layer proteins such as arCOG02884 (a typical representative is MMP0360 from *Methanococcus maripaludis* S2) and their homologs (Fig. 2 and Table 1 and 2). Given that the majority of the components of this hypothetical complex are predicted to be membrane or extracellular proteins, it appears most likely that the complex is a novel secretion system (as opposed to one involved in vesicle formation). This prediction is in line with the recent identification of many unrelated types of the secretion systems in bacteria and the link of arCOG01314 to the gliding motility apparatus that is now considered a distinct secretion system as well [34,36]. Based on the predicted domain architectures, localization and topologies of the constituent proteins (Table 2), and symmetry considerations, we propose a tentative model of this new secretion system (Fig. 2B).

### 3.3. arCOG01994 and its homologs implicated in membrane remodeling or vesicle formation

**Fig. 3:**
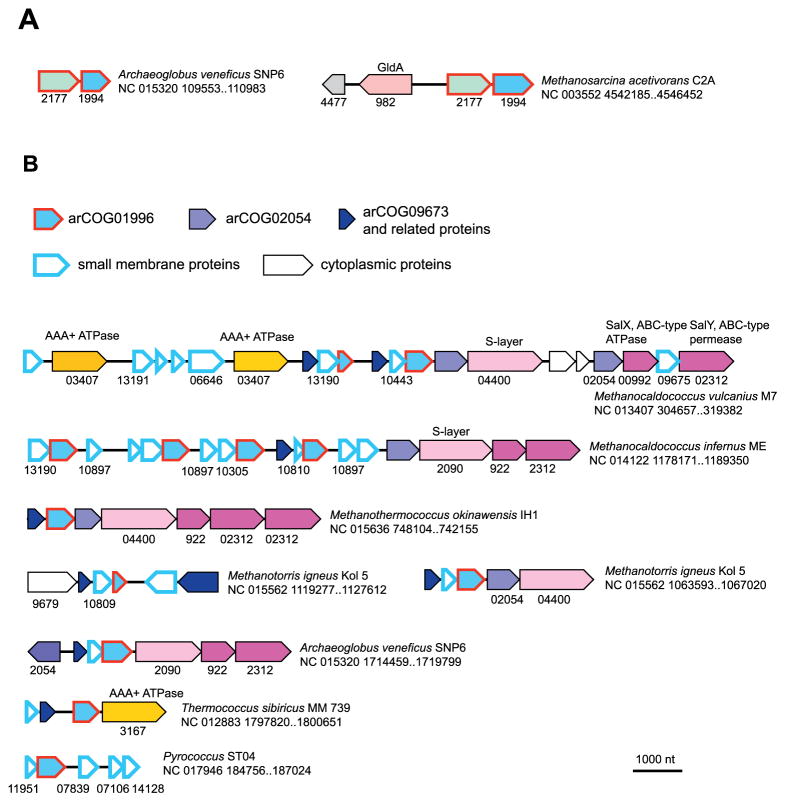
Genomic neighborhood analysis of arCOG01994 and arCOG01996, putative components of membrane remodeling systems. A. Gene neighborhood for arCOG01994. B. Gene neighborhood for arCOG01996. Designations are as in Fig. 1. Respective arCOG numbers are indicated underneath the arrows.

The arCOG01994 (a typical representative is MMP1596 from *M. maripaludis* S2) is represented by several paralogous proteins in many archaea; these proteins are known as COG1300 or pfam01944 (Supplementary Table 2). Altogether, this protein family is present in all major archaeal lineages (Table 1) and is widespread in bacteria as well. The only characterized family member is SpoIIM from *Bacillus subtilis*, a single-copy gene that is involved in sporulation as a subunit of the DMP complex that is responsible for the engulfment of the forespore [43,44]. The membrane protein SpoIIM initiates the assembly of this complex at the septal membrane and recruits the autolysins SpoIIP and SpoIID that jointly degrade peptidoglycan in the forespore wall [43–45]. No homologs of SpoIID or SpoIIP were detected in archaea. The SpoIIM protein is also encoded by many bacteria that do not form spores [46] and thus appears to perform a different function. In spore-forming bacteria, *spoIIM* gene is rarely found in a predicted operon or even within the same locus with other genes involved in sporulation. In several other bacteria, SpoIIM homologs are encoded in a conserved neighborhood with a vWA domain-containing protein and an AAA+ ATPase of COG0714 but never with other surface proteins. In archaea, arCOG01994 proteins are mostly encoded by stand-alone genes. However, several representatives of at least four major euryarchaeal lineages possess a predicted operon that consists of arCOG01994 and arCOG02177 (which also has been projected to LACA, see Table 1 and Supplementary Table 3). The arCOG02177 (a typical representative is MMP0328 from *M. maripaludis* S2) includes highly conserved proteins with 7 predicted TMs and a pair of conserved glutamate and aspartate residues in the loops between the TMs suggesting that these proteins might possess enzymatic activity. In Methanomicrobia, these two genes appear to belong to the same operon with the genes encoding glycerol dehydrogenase GldA (arCOG00982), which is responsible for the synthesis of glycerol-1P, a component of archaeal lipids [47,48], and another uncharacterized protein from the LACA gene set, arCOG04477 (COG1860, pfam03684, a typical representative is MMP1679 from *M. maripaludis* S2), which is present exclusively in archaea and contains two Fe–S clusters (Fig. 3).

Many archaea also possess other paralogs of COG1300 that form at least three more distinct families (Table 1). Among these, arCOG01996 is perhaps the most notable one because it belongs to complex gene neighborhoods (Fig. 3 and Supplementary Table 3) some of which have been described recently in the course of our systematic analysis of the archaeal genomic “dark matter” [49]. Here we add some details to that brief analysis. As shown previously, arCOG01996 genes belong to three- or four-gene cassettes with other genes that encode small, highly diverged membrane proteins (in *Methanocaldococcus vulcanius*, this cassette is typified by three genes: Metvu_0322, Metvu_0323 and Metvu_0325 from arCOG02054, arCOG01996, arCOG09673, respectively). The arCOG02054 proteins typically have 4 predicted TMs and belong to the COG2881 (pfam04893) family which is represented also in bacteria and eukaryotes. One of these proteins, Yip1p, has been shown to be essential in yeast where it promotes Rab GTPase-dependent vesicle formation in the endoplasmic reticulum and Golgi complex and interacts with other membrane proteins, such as Yos1p, a two TM small protein, and Yif1p, a paralog of Yip1p [50,51]. arCOG09673 includes proteins with 3 predicted TMs that belong to an apparently fast-evolving superfamily along with multiple other distantly related arCOGs and possibly numerous other small membrane proteins that are present in respective loci but show no detectable similarity to other proteins (Supplementary Table 3). So far this complex or some parts of it were identified in several representatives of 3 major euryarchaeal lineages (Archaeoglobi, Methanococci, Thermococci), although individual components are more broadly distributed in archaea (Supplementary Table 2). In addition to genes encoding the conserved and diverged, small membrane proteins, these loci include genes coding for other potential components of the same system, such as an AAA+-type ATPase, an ABC-type transporter of the SalXY family, and S-layer proteins (Fig. 3).

Taken together, the observations presented here seem to suggest that arCOG01994 members and their homologs belong to protein complexes that are involved in membrane remodeling and/or vesicle formation. Given that arCOG01994 together with arCOG02177 are present in the majority of the archaea, especially in most of those with FtsZ-based cell division [41], these proteins might play some role in division as well.

### 3.4. arCOG00899, its homologs, and arCOG02245 implicated in Slayer protein glycosylation and lipid metabolism

arCOG00899 represents a vast family of integral membrane proteins (pfam03706, UPF0104, a typical representative is HVO_1859 from *Haloferax volcanii*) of unknown function, with homologs both in archaea and in bacteria (COG0392). While most members of this family are stand-alone proteins, some of them are domains of composite, two-domain proteins which has led to many cases of misannotation. In archaea, many UPF0104 members form C-terminal domains of the AglD family proteins (such as HVO_0798 from *H. volcanii*) that catalyze N-glycosylation of the S-layer glycoprotein and flagellin [52,53]. However, the glycosyltransferase activity has been shown to reside in the N-terminal cytoplasmic domain of AglD [54,55], which left its C-terminal membrane domain (arCOG00899) without a defined function. In bacteria, members of COG0392 often form two-domain combinations with the pfam09924 (DUF2156) domain. In *B. subtilis* and *Staphylococcus aureus*, such two-domain proteins are referred to as MprF (multi-peptide resistance factor) and participate in membrane lipid biosynthesis, catalyzing transfer of the lysyl moiety from Lys-tRNA^Lys^ to phosphatidylglycerol [56–58]. It has been shown that the lysylphosphatidylglycerol synthetase activity of MprF resides in its C-terminal pfam09924 domain [59]. This finding is consistent with the structure of this domain, which has been solved at the Midwest Center for Structural Genomics and is suggestive of N-acyltransferase activity (PDB: 2HQY; Nocek, B., Borovilos, M., Abdullah, J., and Joachimiak, A., unpublished). In contrast, the N-terminal arCOG00899-related domain of MprF is required for the translocation of lysylphosphatidylglycerol from the inner to the outer leaflet of the membrane, suggesting that it has the phospholipid flippase activity [60–62]. In archaea, this gene is encoded in a conserved neighborhood with arCOG00563 (e.g., MJ_1079 from *Methanocaldococcus jannaschii*), an enzyme distantly related to oligosaccharyl transferase of PMT/STT3 family, and arCOG00897 (e.g., MJ_1080 from *M. jannaschii*), AglD-like glycosyltransferase, which is involved in the addition of the terminal hexose of the pentasaccharide [63]. In *H. volcanii*, HVO_2052 (arCOG00563) is encoded within a neighborhood containing genes of the second N-glycosylation pathway [63] although the exact function of HVO_2052 remains unknown. Another genomic context typical of arCOG00899 encompasses arCOG02876 and arCOG01403 (e.g., MA0797 and MA0798, respectively, from *Methanosarcina acetivorans*), a polysaccharide deacetylase family enzyme and a glycosyl-transferase, respectively (Supplementary Table 3). Taken together, these observations strongly suggest that arCOG00899 proteins are involved in archaeal glycosylation pathways. Furthermore, it seems likely that all members of COG0392 possess flippase activity and catalyze translocation of lipid and/or S-layer glycoprotein saccharide to the outer leaf of the cytoplasmic membrane.

An HHpred search started from *M. jannaschii* protein MJ_0933, a representative of arCOG02245 (COG1836, pfam01940), identifies sequence similarity with pfam01148, cytidylyltransferase family, with the probability of 97.57%. Moreover, the two aspartate residues involved in binding the Mg^2+^–K^+^ di-metal center that is essential for the catalytic mechanism in cytidylyltransferase [64] is conserved in arCOG02245 proteins. Several arCOG02245 domain-containing proteins, such as Mbar_A3310 from *Methanosarcina barkeri*, contain another, N-terminal domain that belongs to the same family although more similar to dolichol kinase Sec59. For example, HHpred search started from the first 210 aa of this protein identifies similarity with COG0170, SEC59 Dolichol kinase domain, with the probability of 99.85%. In this domain, the two aspartates presumably involved in catalysis are also conserved. Two enzymes of the cytidylyltransferase family have been characterized as essential enzymes of lipid biosynthesis: CDP-diglyceride synthetase CdsA (COG0575, arCOG04106, e.g., HVO_0332 from *H. volcanii*) and dolichol kinase (arCOG01881, e.g., HVO_0053 from *H. volcanii*). Both of these arCOGs were mapped to LACA and show an overlapping phyletic pattern with arCOG02245. Thus, it seems unlikely that these enzyme have the same specificities. The single conserved context for arCOG02245 genes includes the gene for undecaprenyl pyrophosphate synthase *uppS* (arCOG01532, e.g., HVO_2318 from *H. volcanii*, Supplementary Table 3) which catalyzes the successive condensation of the isopentenyl diphosphate (IPP) molecule to farnesyl diphosphate (FPP) to form long-chain polyprenyl diphosphates [65]. This strong link suggests that arCOG02245 proteins might be involved in the pathway of isoprenoid biosynthesis, in a step requiring kinase activity.

### 3.5. arCOG03426, arCOG03427, arCOG04354, and arCOG04469 include putative transporters

The arCOG04354 (pfam04165 or COG1906, a typical representative is PH0014 from *Pyrococcus horikoshii*) is present only in thermophiles, although not in all of them (Supplementary Table 2). An HHpred search (with another arCOG04354 member, Tneu_1186 from *Pyrobaculum neutrophilum*, used as the query) reveals significant similarity with a variety of substrate/proton or sodium symporters of the tripartite tricarboxylate transporter (TTT) class superfamily including pfam02447, gluconate transporter GntP (probability 99.69%), pfam06808 DctM-like 4-dicarboxylate transporters (probability 99.36%), and other transporter families of this class. The TTT class includes ATP-independent transporters that consist of two membrane subunits and an extracellular solute-binding subunit [66].

The arCOG04469 (COG1784, pfam01970, a typical representative is HVO_0611 from *H. volcanii*) is a euryarchaea-specific protein family that belongs to the TctA transporter family which is also a member of TTT class [67,68]. TctA is a component of the tripartite transporter TctABC, which consists of the extracytoplasmic tricarboxylate-binding receptor TctC and two integral membrane proteins, TctA and TctB [67]. In some Halobacteria and Thermococci (e.g., in the HacjB3_03065-HacjB3_03075 locus of *Halalkalicoccus jeotgali*), the *tctA* gene is located in a neighborhood with *tctB* (arCOG10722) and *tctC* (arCOG13681) but in most archaea arCOG04469 members are encoded by stand-alone genes (Supplementary Table 3). It has been suggested that TctA is the main component of the transporter and might function without the TctB and TctC subunits [66]. Thus, both arCOG04354 and arCOG04469, which have nearly complementary phyletic patterns (Table 1), could be carboxylate transporters. Two additional TTT class transporter components, arCOG01801 and arCOG01906, that are reconstructed to LACA (Supplementary Table 1) and typically are encoded in the same locus (data not shown) also might be involved in carboxylate transport. In these arCOGs there are several representatives of the genomes that are not present in arCOG04354 and arCOG04469.

The arCOG03427 (COG2510, pfam00892, a typical representative is HVO_0625 from *H. volcanii*) and its paralog arCOG03426 (a typical representative is MMP1587 from *M. maripaludis S2*) appear to be short variants of the drug/metabolite (DMT) superfamily transporters [69]. An HHpred search starting from any protein of this family identifies multiple DMT transporters with the probability of 90% and higher. The best match is pfam0892, EamA-like transporter family, which is involved in O-acetyl-serine/cysteine export in *Escherichia coli* [70], but other related transporters have distinct specificities [71]. In this case, genomic context does not provide any further clues.

### 3.6. arCOG04002 and arCOG02078 proteins implicated in electron transfer and energy metabolism

An HHpred search reveals that arCOG04002 (and homologs, pfam14358 or DUF4405) belongs to the cytochrome *b* family. For example, the search started with a typical representative protein Arcpr_0821 from *Archaeoglobus profundus* identifies similarity to profile pfam01292, “prokaryotic cytochrome b561”, with the probability of 96.55%, and many other sequences related to cytochrome *b*. The output alignments cover 2 TMs with two characteristic conserved histidine residues involved in heme binding, so that the archaeal protein represents about half of the cytochrome *b*_561_ molecule. Generally, cytochrome *b* family proteins are involved in electron transport [72,73]. Thermococci possess several paralogous members of this family some of which are encoded in a moderately conserved gene context represented, e.g., by the gene locus CL1_0239 – CL1_0246 from *Thermococcus* CL1. In addition to the arCOG04002 gene, this neighborhood contains genes of arCOG00709 and arCOG01619, aldehyde:ferredoxin oxidoreductase and NADP-dependent oxidoreductase, respectively; two genes of arCOG03957 encoding ferritin-like proteins; transcriptional regulator of the ArsR family (arCOG01679); an uncharacterized secreted protein (arCOG07800), and a secreted protein with a small coiled-coil domain (arCOG08354). In *Thermococcus barophilus*, the arCOG04002 domain is fused to a cytochrome b_5_-like heme binding domain, pfam00173. Thus, predicted functions of these genes are compatible with the hypothesis that arCOG04002 proteins are involved in iron-dependent electron transfer and might be components of multisubunit redox complexes, at least in Thermococci.

The arCOG02078 (COG1852, pfam01976, DUF116, typical representative is MMP0162 from *M. maripaludis S2*) also includes predicted metal-binding proteins. These proteins contain two TMs (the first one might correspond to a signal peptide) at the N-terminus followed by a globular domain with at least six conserved cysteine residues, three of which match the motif CxxCxxC that is characteristic of one of the families of ferredoxins [73]; otherwise, however, the pattern of cysteine conservation in these proteins is distinct. An HHpred search started with Metho_1507 from *Methanomethylovorans hollandica* detects its similarity to a variety of proteins with the receiver (REC) domain, including a hit to the PDB entry 1SRR), sporulation response regulatory protein Spo0F, with the probability of 91.55%. These results suggest that the arCOG02078 domain adopts a flavodoxin-like fold. Analysis of the relevant genomic contexts reveals association of this gene with sulfite or nitrite reductase components in most Methanomicrobia (e.g., the Metho_1504-Metho_1508 locus in *M. hollandica*) and with an oxyanion-translocating ATPase in Methanococci (e.g., the MJ1141–MJ1142 locus in *Methanocaldococcus jannaschii*). In some bacteria, this domain is fused to polyprenyl synthetase (e.g., Psta_0175 from *Pirellula staleyi*). Given that all the neighboring genes are involved in energy conversion, these observations are best compatible with a role of arCOG02078 in electron transfer.

### 3.7. Other ancestral arCOGs that include membrane proteins

The arCOG02008 (COG3371, pfam06197, a typical representative is Saci_1688 from *S acidocaldarius*) family is conserved in diverse archaea but rare in bacteria. As suggested by HHpred search results, arCOG02008 proteins are homologous to eukaryotic proteins of the Frag1/DRAM/Sfk1 family (e.g., the search started with OCC_03592 from *Thermococcus litoralis* identifies pfam10277, Frag1/DRAM/Sfk1 family with the probability of 98.33%). In eukaryotes, these proteins are involved in stress response and lipid metabolism but their exact function is not clear [74,75]. There is no distinct, conserved context associated with these genes in archaeal genomes although they are often encoded next to the ABC-type transporter operons. In Thermococci, this gene is often located near the tRNA S(4)U 4-thiouridine synthase gene [76] suggesting that arCOG02008 could be involved in sulfur transfer. The presence of a strictly conserved histidine is compatible with this hypothesis given that a catalytic histidine is involved in the mechanism of acyl-CoA thioesterases and other sulfur-transfer enzymes [77].

Members of arCOG03206 (COG1822, pfam01901, a typical representative is MMP1111 from *M. maripaludis* S2) and their paralogs are present only in three major euryarchaeal lineages and in *Korarchaeum* (Table 1). Typically, two paralogs are encoded within the same predicted operon together with a gene of arCOG03414, an uncharacterized secreted protein (Supplementary Table 3). Additionally, these three genes often co-localize with arCOG00563 or arCOG00566, both predicted glycosyltransferases of the PMT family (Supplementary Table 3). HHpred identifies significant similarity of the arCOG03206 proteins with O-antigen polysaccharide polymerase Wzy (e.g., a search initiated with MJ0420 from *Methanocaldococcus jannaschii* detects similarity with the profile for pfam14296, O-antigen polysaccharide polymerase Wzy, with the probability of 98.12%), the enzyme responsible for polysaccharide polymerization in the pathway of complex lipopolysaccharide (e.g., lipid A in Proteobacteria) biosynthesis [78]. Several archaeal proteins, including MJ0420, have been annotated as “O-antigen polymerase”. The results of an HHpred search started from Mefer_1301 from *Methanocaldococcus fervens*, which belongs to arCOG03414, reveal significant similarity with a part of the sequence of the sugar-binding domain of the archaeal transcriptional regulator TrmB (probability 99.52%). Thus, arCOG03206, its paralogs, and arCOG03414 are likely to be involved in a lipopolysaccharide biosynthesis and/or glycosylation pathway that is specific for the respective archaea.

The arCOG02159 (COG4089, pfam07758, a typical representative is Saci_0392 from *S. acidocaldarius*) family is the only one of the 15 ancestral arCOGs analyzed here for which only a general functional prediction appeared attainable. This arCOG includes proteins with moderately conserved sequences containing 6 predicted TMs and a relatively large (about 50 amino acids) loop between TM2 and TM3 for which an all-beta secondary structure is predicted. Homologs of these proteins are present also in several bacteria where they are encoded in a moderately conserved gene neighborhood together with Stage II sporulation protein P (SpoIIP, pfam07454) and another uncharacterized gene, *yphB*, which is involved in sporulation in *B. subtilis* (pfam14045, YIEGIA protein). SpoIIP is an enzyme related to the N-acetylmuramoyl-L-alanine amidase (pfam01520) family and in spore-forming bacteria is a subunit of the DMP complex mentioned above in connection with the archaeal SpoIIM homolog. Similarly to SpoIIM, SpoIIP is present in many bacteria that do not form spores. Thus, SpoIIP and by inference arCOG02159 proteins could represent yet another protein complex involved in membrane remodeling.

## 4. Discussion

The above-described analysis of 15 ancestral arCOGs that include predicted membrane proteins led to the identification of the universal protein insertase YidC in the great majority of archaea, identification of several putative secretion and/or membrane remodeling systems, and more general prediction of membrane-associated functions for several other proteins. It should be noted that comparative genomic analysis presented here yielded functional prediction not only for the highly conserved proteins that were used as seeds but also for numerous proteins with which these conserved proteins share genomic neighborhoods. The results of this work, together with our previous observations made using a complementary approach [49], reveal an unexpected diversity of putative membrane-associated functional systems in archaea, in particular those implicated in secretion. The presently known archaeal secretion and membrane remodeling systems might represent the tip of the proverbial iceberg of diverse molecular machines that remain to be characterized experimentally.

The most consequential general conclusion from the present analysis is that the already available collection of archaeal (and bacterial) genomes, albeit only a drop in the bucket of the entire microbial world, seems to be sufficient to obtain specific functional predictions for the great majority – if not all – highly conserved genes and functional systems. Beyond doubt, a long “tail” of rare and highly variable systems will remain uncharacterized for many years to come but the widespread ones appear to be tractable now. Thus, a complete census of such functional modules based on a combination of exhaustive sequence analysis, structure prediction, comparison of phyletic patterns, and genomic neighborhood analysis (the “guilt by association” approach) seems to be both a worthy and a feasible undertaking.

Another observation with a general impact stems from the analysis of YidC, the nearly universal membrane insertase that, until this work, was conspicuously undetectable in Crenarchaeota. A combination of comparative genomics and in-depth protein sequence analysis allowed us to pinpoint the likely orthologs of YidC in nearly all archaea although in some of these organisms, the sequence similarity is near the limit of detection. Such findings “restore” the universality of widespread genes but also indicate that even proteins present in (nearly) all cellular life forms can show surprising level of variation that probably reflects functional differences between the respective systems. Similar observations have been made previously in our analysis of DNA replication [79,80] and cell division [41] systems of archaea. Thus, focused search for highly diverged orthologs of otherwise highly conserved genes, followed by experimental validation, seems to be a promising research program.

## Supplementary Material

Supplemental Table 1

Supplemental Table 2

Supplemental Table 3

## Figures and Tables

**Table 1:** Uncharacterized ancestral arCOGs that include predicted membrane proteins.

arCOG	Related arCOGs	Number of TMs	Presence/absence pattern in major lineages^a^													Prediction/comment
			Desulfu- rococci	Sulfo- lobi	Thermo- protei	Thaumar- chaeota	Korar- chaeota	Archaeog- lobi	Halo- bacteria	Methano- bacteria	Methan- ococci	Methano- microbia	Thermo- cocci	Thermo- plasmata	Nanoar- chaeota	
02673	07287 08873	3,4	p	1	–	1	1	1	1	1	1	1	1	1	1	Sec system component, YidC ortholog (see Fig. 1);
01994	01995 01996 05021	3 or 6	p	1	p	1	1	1	1	1	1	1	1	p	1	Putative membrane remodeling system component (see Fig. 3); Many archaea have multiple paralogs
02177	02178	7	–	–	–	–	–	1	1	p	1	1	1	–	1	Putative membrane remodeling system component (see Fig. 3)
01314		2	p	p	p	–	1	1	p	p	p	p	1	–	–	Putative secretion system component (see Fig. 2,Table 2)
02884	02886 02887 11424	2 or 4	p	p	p	–	–	p	p	p	p	p	P	p	–	Putative secretion system component (see Fig. 2,Table 2)
00899	00901 00903 00902 00898	7–9	p	–	p	1	1	1	1	1	1	1	1	p	–	Flippase involved in the protein glycosylation pathways; many archaea have multiple paralogs
02245		6–14	–	–	1	–	1	1	1	1	1	1	1	p	–	Cytidylyltransferase family enzyme involved in lipid metabolism
04002	08353 11324	2,3	–	–	p	p	1	p	–	–	–	p	P	p	–	Cytochrome *b* superfamily, possibly involved in electron transfer as a component of redox complexes; expanded in several Thermococci
02078		2	–	–	–	–	1	–	–	1	1	1	P	–	–	Possibly involved in electron transfer as a component of redox complexes;
03427	03426	4,5	p	–	–	–	–	–	p	–	1	–	P	p	–	Transporter; expansion in Thermococci
02008		4	1	1	1	–	1	–	p	p	–	–	1	1	–	Transporter component
04354		12	p	–	p	–	1	1	–	–	–	–	P	–	–	Transporter
04469		11	–	–	–	–	–	p	1	1	1	1	1	p	1	Transporter
02159		7	p	1	p	–	–	1	–	–	–	1	p	p	–	No prediction; expansion in Methanomicrobia
03206	03207 05723	8–10	–	–	–		1	–	–	p	1	–	1	–	–	Lipopolysaccharide biosynthesis

a Complete phyletic patterns are provided in the Supplementary Table 2.

The patterns are abbreviated as follows:–, absent in the vast majority of the respective genomes; p, partially present, and 1, present in the vast majority of genomes.

**Table 2:** Putative novel secretion system associated with arCOG01314.

arCOG and its homologs	Domain architecture and predicted activity
arCOG01314	Membrane-anchored protein with extracellular flavodoxin-like domain
arCOG00435 arCOG00434	AAA + ATPase of MoxR/GvpN family, possibly involved in the complex assembly and regulation of its state
arCOG02742 arCOG02747	Likely secreted protein with an N-terminal Ig like domain fused to the C-terminal von Willebrand factor type A (vWA) domain
arCOG02487 arCOG12973 arCOG02488	Predicted S-layer protein with a Ig-like domain
arCOG02884 arCOG02887 arCOG11424	Membrane-associated predicted S-layer protein with an extracellular Ig-like domain; 2 or 4 TMs; belongs to COG4743, pfam07760
arCOG03442 arCOG11910	Membrane-associated predicted S-layer protein with an extracellular Ig-like domain; 2 or 4 TMs; belongs to COG4743, pfam07760
arCOG03875 arCOG05098 arCOG10494	Membrane protein with 2–3 TMs
arCOG07126	Membrane protein with 5 TMs
arCOG11232	Membrane protein with 5 TMs

## Appendix A. Supplementary data

Supplementary data related to this article can be found at http://dx.doi.org/10.1016/j.biochi.2015.01.004.
